# Supplementary material for: Novel Fluticasone Propionate and Salmeterol Fixed-Dose Combination Nano-Encapsulated Particles Using Polyamide Based on L-Lysine
Source: Pharmaceuticals (Basel). 2022 Mar 8;15(3):321. doi: 10.3390/ph15030321 (PMC8955190; doi:10.3390/ph15030321)
Supplement: Supplementary file 1 [file pharmaceuticals-15-00321-s001.zip › pharmaceuticals-1607491-supplementary.pdf]

pharmaceuticals

MDPI

Supplementary Materials

Novel Fluticasone Propionate and Salmeterol Fixed-Dose Combination Nano-Encapsulated Particles Using Polyamide Based on L-Lysine

Mohammad H. Alyami[1], Eman Zmaily Dahmash[2], Dalia Khalil Ali[3], Hamad S. Alyami[1,] *, Hussien AbdulKarim[2], and Samar A. Alsudir[4]

[1] Department of Pharmaceutics, College of Pharmacy, Najran University, Najran, Kingdom of Saudi Arabia; mhalmansour@nu.edu.sa, hsalmukalas@nu.edu.sa

[2] Department of Applied Pharmaceutical Sciences and Clinical Pharmacy, Faculty of Pharmacy, Isra University, Amman, Jordan; eman.zmaily@iu.edu.jo, hussein.abdalkarem@yahoo.com

[3] Department of Physiotherapy Department, Faculty of Allied Medical Sciences, Isra University, Amman, Jordan; dalia.ali@iu.edu.jo

[4] National Center of Biotechnology, Life Science and Environmental Research Institute, King Abdulaziz City for Science and Technology, Riyadh, Saudi Arabia; salsadeer@kacst.edu.sa

* Correspondence: Hamad S. Alyami, email: hsalmukalas@nu.edu.sa , Tel.: +966500095255

Section S1

Particle size analysis

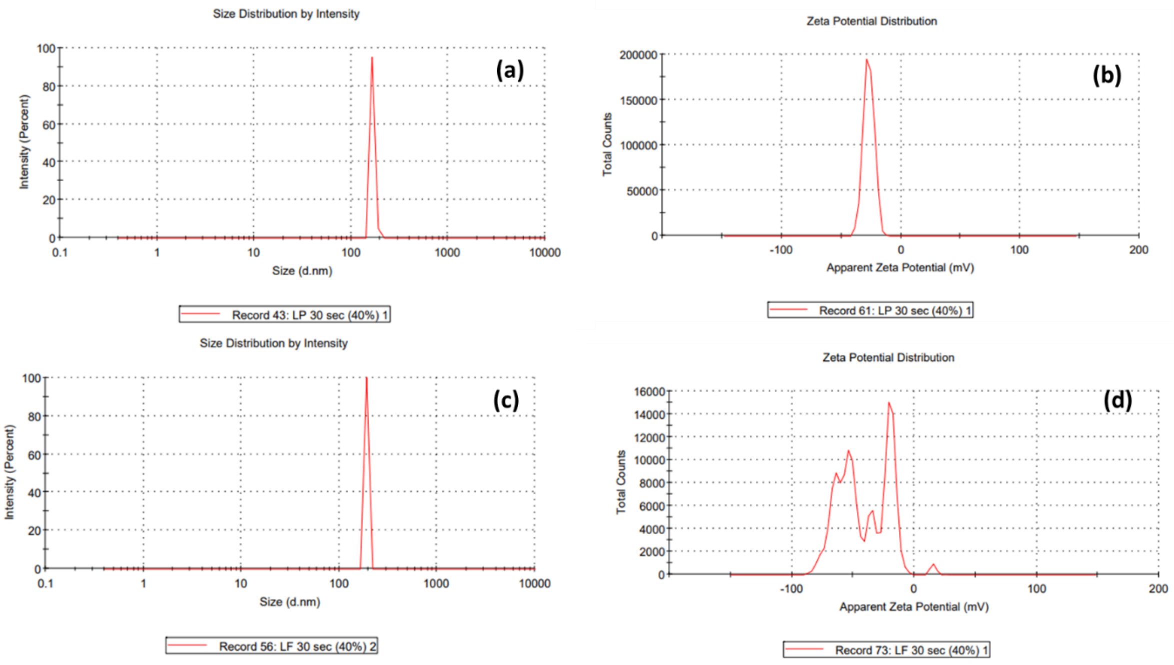

Figure S1. Representative images of particle size analysis (PSA) and zeta potential for (a) PSA of (FP-SAL/Lys-PA) NCs, (b) zeta potential of (FP-SAL/Lys-PA) NCs, (c) PSA of of Lys-PA NCs and (d) zeta potential analysis for Lys-PA NCs.

Section S2

Release Kinetic Modelling

Figures S2-S6 demonstrated the linear regression of the five release kinetics mathematical models for fluticasone propionate (FP).

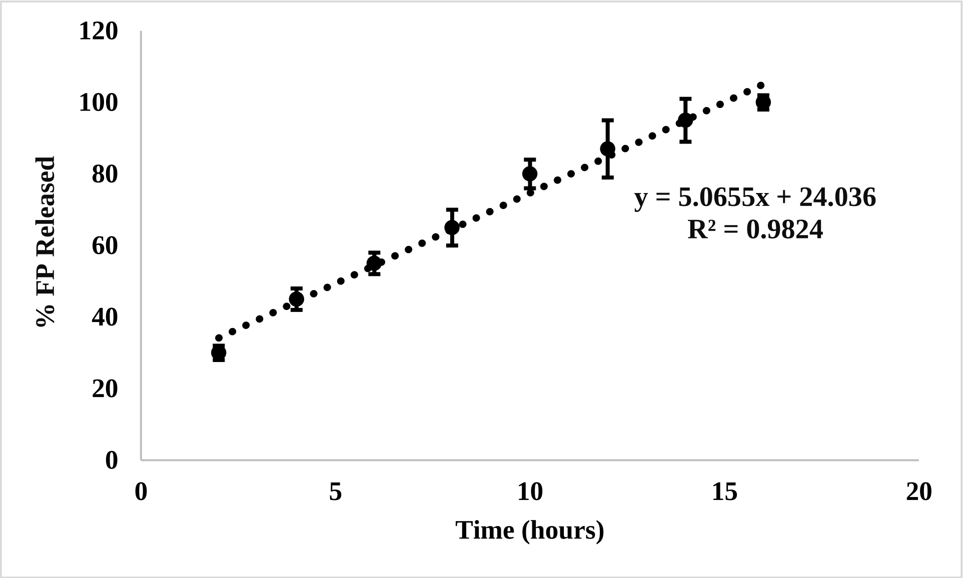

Figure S2. Linear regression of Zero Order Kinetics mathematical model of FP from FP-SAL-loaded Polyamide based on L-Lysine nanocapsules (F3) with the related equation, and coefficient of determination (R^2) value.

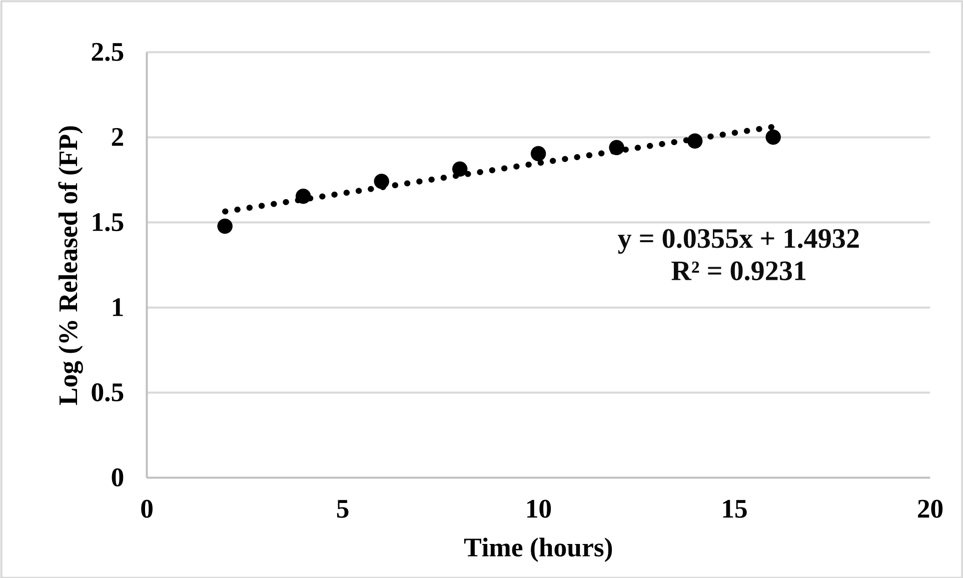

Figure S3. Linear regression of First Order Release Kinetics mathematical model of FP from FP-SAL-loaded Polyamide based on L-Lysine nanocapsules (F3) with the related equation, and coefficient of determination (R^2) value.

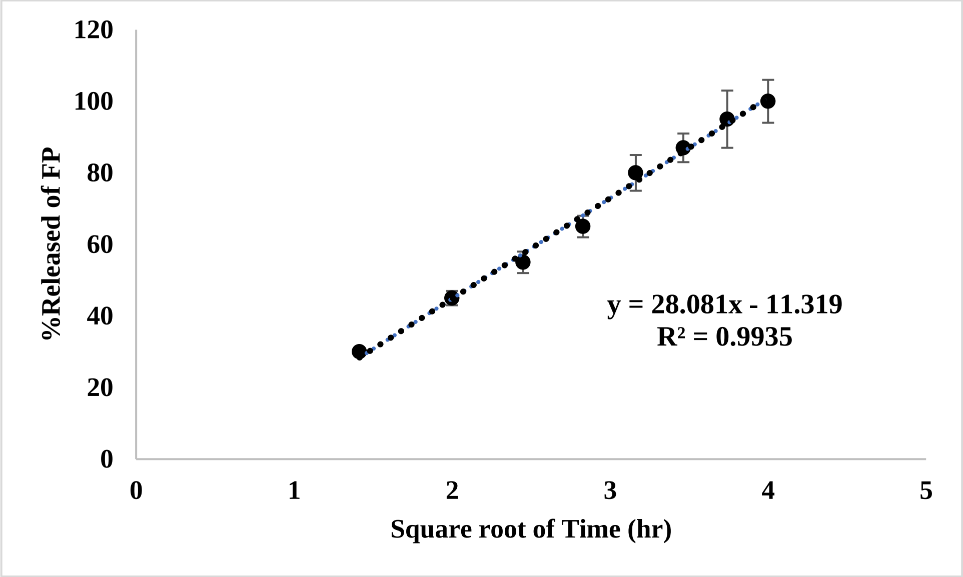

Figure S4. Linear regression of Higuchi Release Kinetics mathematical model of FP from FP-SAL-loaded Polyamide based on L-Lysine nanocapsules (F3) with the related equation, and coefficient of determination (R^2) value.

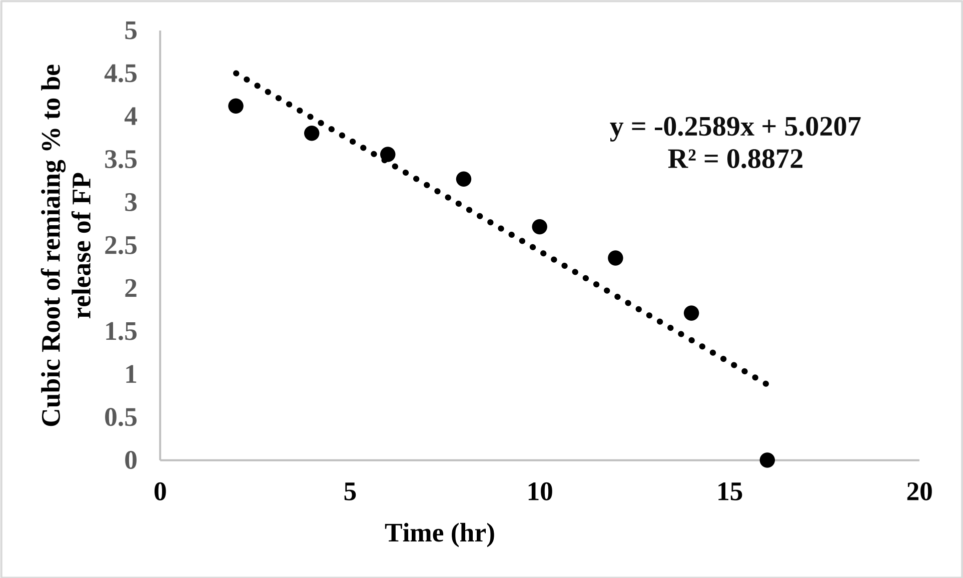

Figure S5. Linear regression of Hixon Crowell Release Kinetics mathematical model of FP from FP-SAL-loaded Polyamide based on L-Lysine nanocapsules (F3) with the related equation, and coefficient of determination (R^2) value.

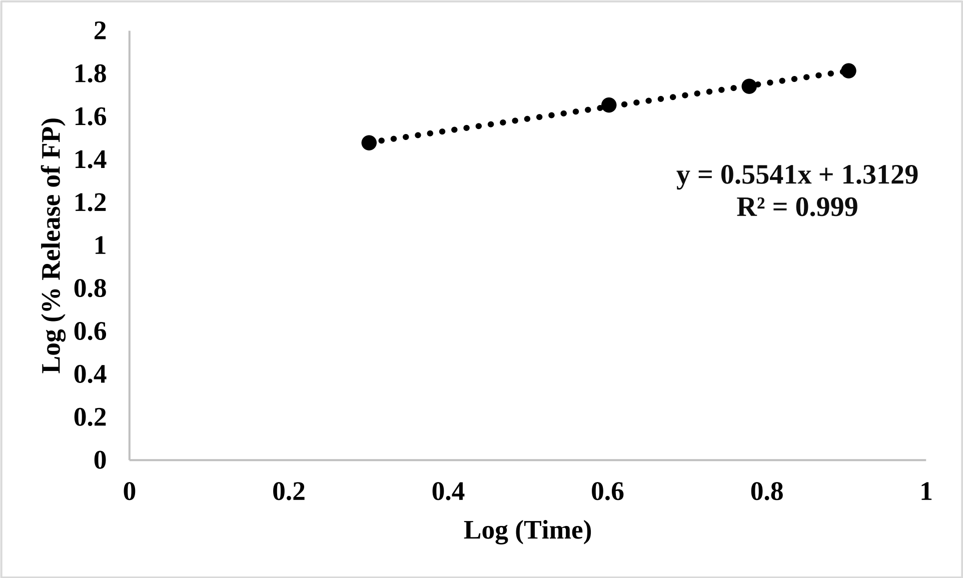

Figure S6. Linear regression of Korsmeyer -Peppas Release Kinetics mathematical model of FP from FP-SAL-loaded Polyamide based on L-Lysine nanocapsules (F3) with the related equation, and coefficient of determination (R^2) value.

Figures S7-S11 demonstrated the linear regression of the five release kinetics mathematical models for salmeterol xenofoate (SAL).

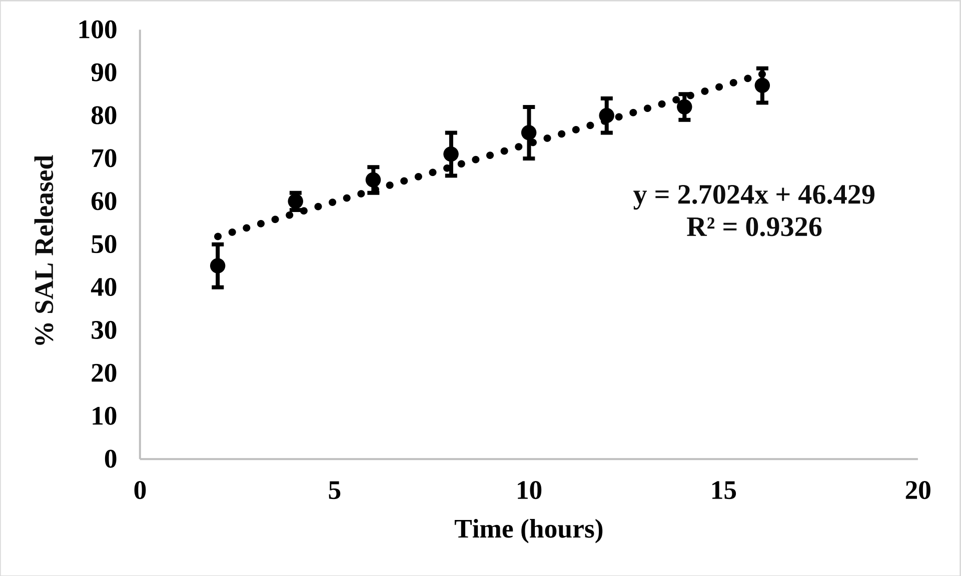

Figure S7. Linear regression of Zero Order Kinetics mathematical model of SAL from FP-SAL-loaded Polyamide based on L-Lysine nanocapsules (F3) with the related equation, and coefficient of determination (R^2) value.

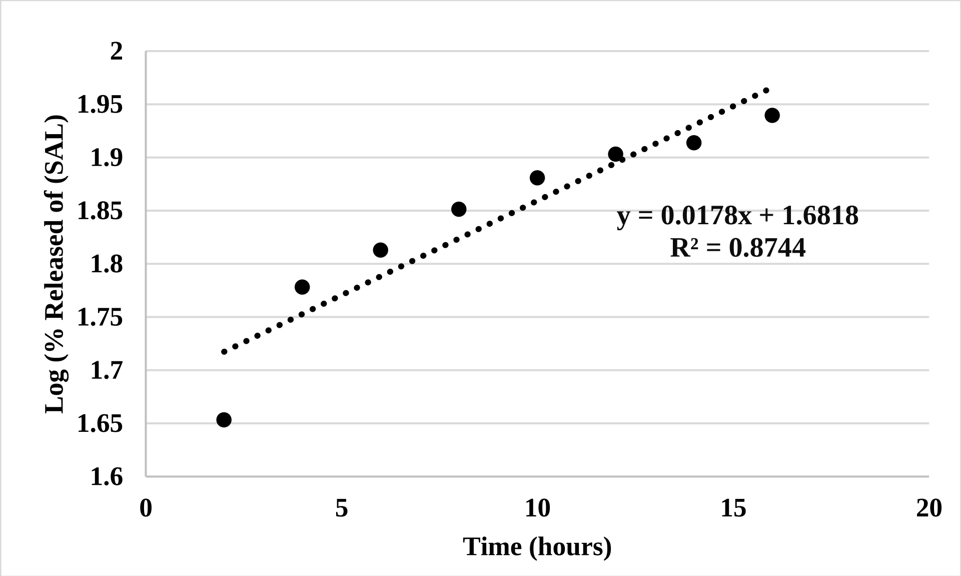

Figure S8. Linear regression of First Order Release Kinetics mathematical model of SAL from FP-SAL-loaded Polyamide based on L-Lysine nanocapsules (F3) with the related equation, and coefficient of determination (R^2) value.

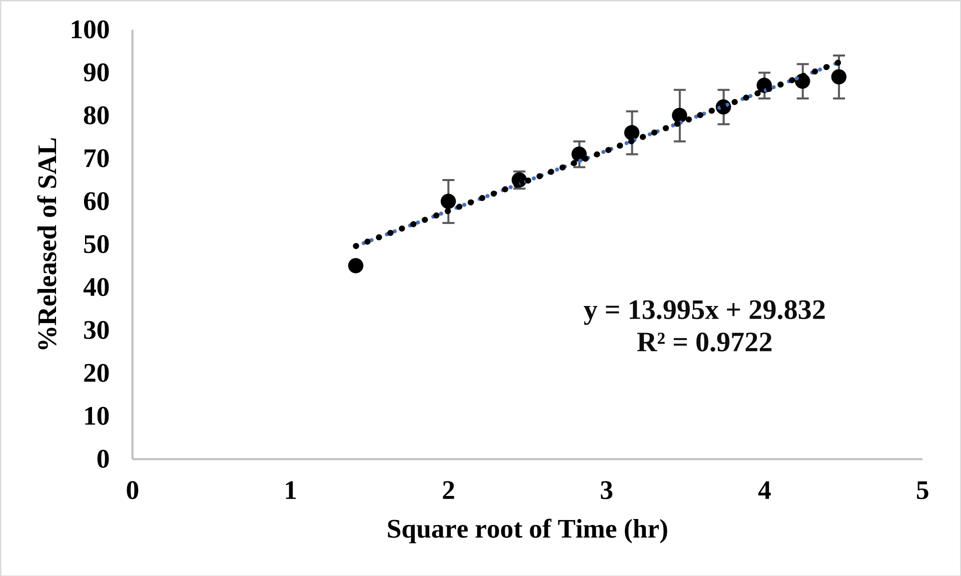

Figure S9. Linear regression of Higuchi Release Kinetics mathematical model of SAL from FP-SAL-loaded Polyamide based on L-Lysine nanocapsules (F3) with the related equation, and coefficient of determination (R^2) value.

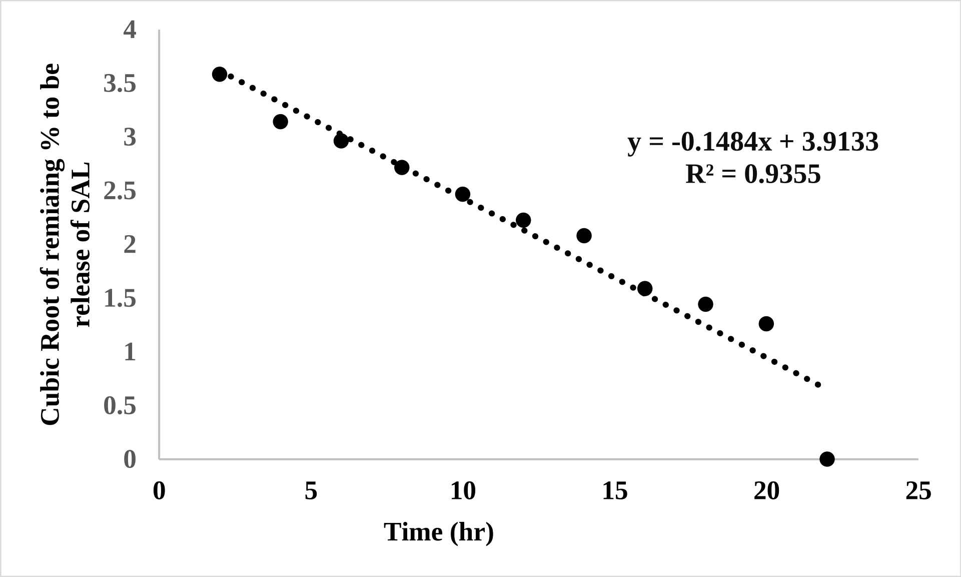

Figure S10. Linear regression of Hixon Crowell Release Kinetics mathematical model of SAL from FP-SAL-loaded Polyamide based on L-Lysine nanocapsules (F3) with the related equation, and coefficient of determination (R^2) value.

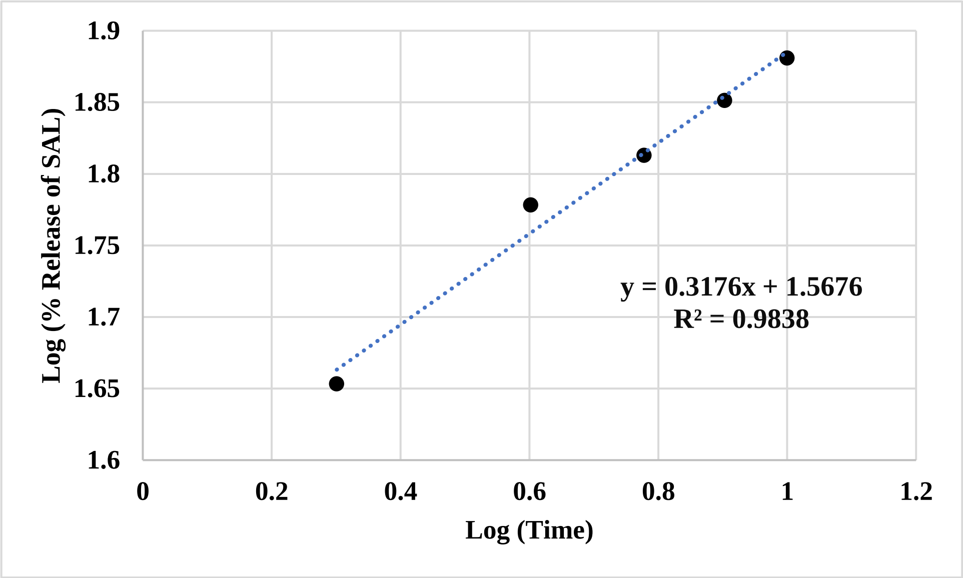

Figure S11. Linear regression of Korsmeyer -Peppas Release Kinetics mathematical model of SAL from FP-SAL-loaded Polyamide based on L-Lysine nanocapsules (F3) with the related equation, and coefficient of determination (R^2) value.

Section S3

SEM images

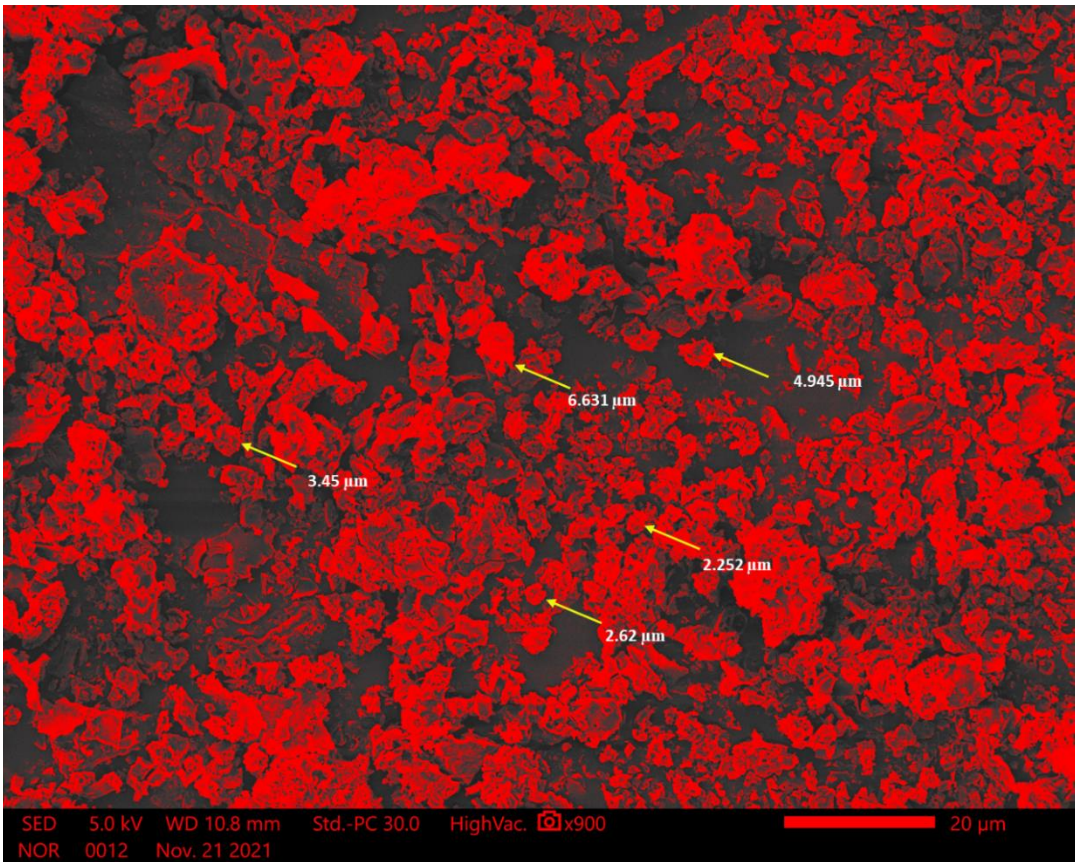

Figure S12. SEM micrographs of (FP-SAL/Lys-PA) NCs at 900 X magnification after image adjustment into coulor threshold using ImageJ software. Arrows highlight representative aggregates that were measured for particle size analysis.
